# A Fast Circle Detection Algorithm Based on Information Compression

**DOI:** 10.3390/s22197267

**Published:** 2022-09-25

**Authors:** Yun Ou, Honggui Deng, Yang Liu, Zeyu Zhang, Xusheng Ruan, Qiguo Xu, Chengzuo Peng

**Affiliations:** School of Physics and Electronics, Central South University, Lushan South Road, Changsha 410083, China

**Keywords:** circle detection, information compression, average sampling verification

## Abstract

Circle detection is a fundamental problem in computer vision. However, conventional circle detection algorithms are usually time-consuming and sensitive to noise. In order to solve these shortcomings, we propose a fast circle detection algorithm based on information compression. First, we introduce the idea of information compression, which compresses the circular information on the image into a small number of points while removing some of the noise through sharpness estimation and orientation filtering. Then, the circle parameters stored in the information point are obtained by the average sampling algorithm with a time complexity of O(1) to obtain candidate circles. Finally, we set different constraints on the complete circle and the defective circle according to the sampling results and find the true circle from the candidate circles. The experimental results on the three datasets show that our method can compress the circular information in the image into 1% of the information points, and compared to RHT, RCD, Jiang, Wang and CACD, Precision, Recall, Time and F-measure are greatly improved.

## 1. Introduction

Accurately obtaining information about circles in images has always been a difficult and important problem in computer vision. At present, circle detection is widely used in spacecraft [1], industrial component measurement [2], pupil positioning [3], medical image analysis [4], circular traffic sign detection [5], Blast-Hole Detection [6] and other fields. With the continuous increase in application fields, people put forward higher requirements for the performance of circle detection algorithms.

Hough Transform [7] is the most classical circle detection algorithm. The basic idea is to transform the original image data into the parameter space and vote for each point. This method is insensitive to noise and has strong robustness, but the algorithm needs to vote on any three points in the parameter space, which requires high time and space. In order to solve the time defect of the HT algorithm, Xu et al. [8] proposed the Randomized Hough Transform (RHT). This method randomly selects three points to calculate circle parameters for voting and retains circle parameters that reach a certain threshold. Compared with HT, the RHT algorithm has some progress in time, but the memory requirements are still very high. To reduce the memory requirement, Chen et al. [9] proposed a random circle detection algorithm (RCD). RCD samples one more point than RHT and uses a fourth point to replace the linked list of parameters in the RHT algorithm [10]. Therefore, compared to RHT, RCD reduces a lot of memory consumption, but, compared with the randomly sampled three points of the RHT algorithm, the probability of randomly sampling four points on the same circle is very low. Therefore, the sampling efficiency of RCD is very low. In view of this shortcoming, Jiang et al. [11] proposed a method based on difference region sampling. When a candidate circle is determined to be a false circle, if the number of points on the candidate circle reaches a certain value number, a certain number of samples are drawn from its difference area. This method improves the sampling efficiency and has a certain improvement in time compared to RCD. However, if there are small edges around the false circle, there will be more points in the collection area of difference evidence, and the time consumed by the algorithm will increase sharply. Different from Jiang, Wang et al. [12] proposed a sub-pixel circle detection algorithm, which only needs to randomly sample one edge point and then sample according to the gradient rule of the edge point. The algorithm has some improvements in time, but once random noise appears in the image, the accuracy of the algorithm degrades rapidly, so the algorithm does not perform well in real-world images. This random sampling-based algorithm only needs one correct sampling to find the true circle, which often has strong robustness [10] and has a good tolerance for noise. However, this kind of algorithm has a very low probability of correct sampling and often needs to use an algorithm with a time complexity of $O\left(n^{2}\right)$ to screen candidate circles, which results in a serious time-consuming algorithm, and this kind of algorithm generally cannot verify the defect circle.

The main reason for the time-consuming methods of the above random classes is the large number of point iterations and traversal computations [13]. To solve this problem, another method is to connect the edge points in the image into a curve and then obtain the circle parameters through the information analysis of the curve. Le et al. [14] used a line segment detector [15] to extract circular curves, followed by least squares fitting to obtain circular parameters. Although this method achieves good performance, it also suffers from the problem of useless least squares fitting and redundant computation caused by straight lines [16], resulting in longer detection times. Different from Le, Zhen et al. [17] proposed a circle detection algorithm based on the curvature of the edge, which estimated the circle parameters through the curvature and performed hierarchical iterative screening of the radius, but the radius layer of the algorithm needed to be preset, and a lot of time is wasted for larger images. This algorithm of connecting edge points into a curve tends to run faster and exhibits better performance for images with clear and continuous edges, but it is very dependent on the edge extraction results, and for some edges with a large number of edge curves crossing each other or discontinuous edges, the image performance is often poor. We summarize the previous work in Table 1.

In order to improve the speed and accuracy of the random sampling stage in circle detection, reduce the time complexity and memory consumption of the candidate circle screening algorithm and ensure good performance for discontinuous edges and complex curves, this paper proposes a fast circle detection algorithm based on information compression. First, we compress the circle information on the image into information points and then delete some interference information. Then, we use an average sampling algorithm with a time complexity of O(1) to filter out candidate circles and finally verify the complete circle and defect circle, respectively. The experimental results show that the algorithm has the characteristics of high speed, high precision and strong robustness.

The main contributions of this paper are as follows:

(1) Proposing a method to record the information of the inner circle of the image with a few points (information points) and to remove some noise in the image according to the information points.

(2) Proposing an average sampling candidate circle verification method with a time complexity of O(1) and a verification method for defect circles.

The rest of the paper is organized as follows: Section 2 presents our circle detection principle, Section 3 presents the algorithm flow and pseudocode, Section 4 presents the running results of the algorithm and the threshold analysis of parameters and Section 5 concludes the paper.

## 2. Principles of Circle Detection

Our proposed circle detection algorithm consists of four stages: image preprocessing, information compression and filtering, average sampling to verify candidate circles and finding true circles.

### 2.1. Image Preprocessing

In order to smooth the image and reduce the impact of noise on subsequent algorithms [10,11,12,13,14,16,17], we first perform Gaussian filtering on the image. Then, we use an adaptive canny edge extraction algorithm [18] to obtain edges. After edge extraction, we connect adjacent edge points into arc point sets. Note that if the endpoints of two arc point sets are not more than one pixel apart, they are to be merged into the same arc point set. Arc point sets smaller than λ (in this paper, λ=30) pixels are considered to be caused by noise or unimportant details and should be removed [16,17,19]. In our method, the value of parameter λ does not depend on factors such as image size, noise, etc. In each arc point set, we use the method in reference [20] to roughly estimate the sharpness transformation on the curve, which is calculated as follows:

(1) $$R\left(P_{i}\right)=\frac{d_{1}}{d_{2}+d_{3}}$$

where:

(2) $$d_{1}=\sqrt{{\left(PX_{i-k}-PX_{i+k}\right)}^{2}+{\left(PY_{i-k}-PY_{i+k}\right)}^{2}}$$

(3) $$d_{2}=\sqrt{{\left(PX_{i}-PX_{i-k}\right)}^{2}+{\left(PY_{i}-PY_{i-k}\right)}^{2}}$$

(4) $$d_{3}=\sqrt{{\left(PX_{i}-PX_{i+k}\right)}^{2}+{\left(PY_{i}-PY_{i+k}\right)}^{2}}$$

**Figure 1:**
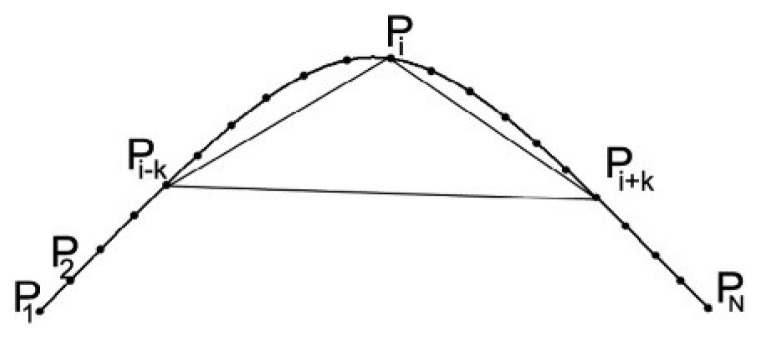
The curvature estimation measurement algorithm we use.

In Figure 1, $P_{1}-P_{N}$ is the points on the curve, and $PX_{i}\;\mathrm{and}\;PY_{i}$ refer to the abscissa and ordinate of the i point, respectively. $PX_{i+k},PY_{i+k},PX_{i-k},PY_{i-k}$ are to move *k* pixels forward and backward, respectively, and $R\left(P_{i}\right)$ refers to the sharpness of the point.

Traverse the points in each edge set, find the points with a similar sharpness and record them. When the points meet the condition (5), they are considered to have a similar sharpness:

(5) $$\{\begin{array}{c} R\left(P_{i}\right)\neq 1\\ R\left(P_{i-1}\right)\neq 1\\ abs\left(R\left(P_{i}\right)-R\left(P_{i-1}\right)\right)<0.2 \end{array}$$

**Figure 2:**
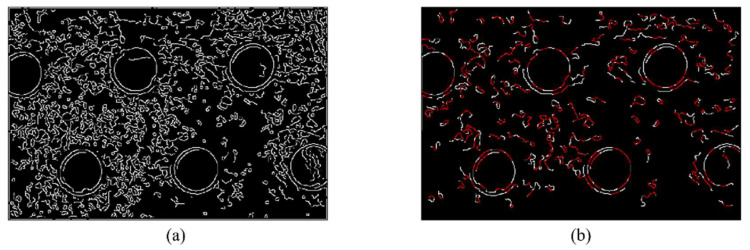
Intermediate results obtained by the algorithm: (**a**) the result of adaptive canny edge extraction, (**b**) the image after sharpness estimation. The recorded arcs are marked in red.

When $R\left(P_{i}\right)=1$, the selection is ended. If the length of the arc at this time is greater than L (see 4.1 for parameter analysis), record this arc. In Figure 2, (a) is the image after removing arcs with lengths less than 30, and (b) is the image after sharpness estimation. The recorded arcs are marked in red.

### 2.2. Information Compression and Filtering

#### 2.2.1. Definition of Information Point

**Figure 3:**
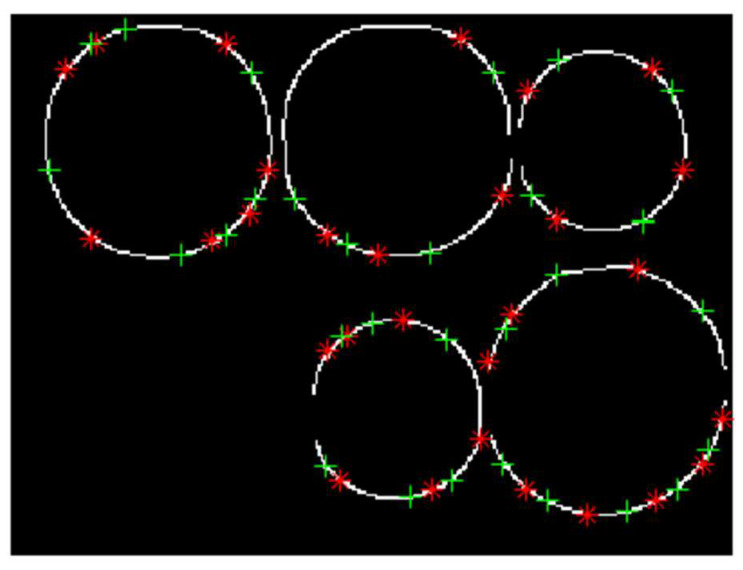
The distribution of information points on the image, where ‘*’ represents information point calculation parameters, and ‘+’ represents information point verification parameters.

For convenience, the definitions of information points are given here. In the image, the information point is the point used to store the circle information on the image. It contains the coordinates of two points, one of which is called the information point calculation parameter, which is used to calculate the circle parameter, and the other is called the information point verification parameter, which is used to validate circle parameters. As shown in Figure 3, ‘*’ represents the information point calculation parameter, which is used to calculate the circle parameter; ‘+’ represents the information point verification parameter, which is used to verify the circle parameter. ‘*’ and ‘+’ are located at both ends of the arc marked in the sharpness estimation, respectively, and the circle information can be obtained by calculating parameters from any three information points on the same circle.

#### 2.2.2. Selection of Information Points and Deletion of Interference Information

**Figure 4:**
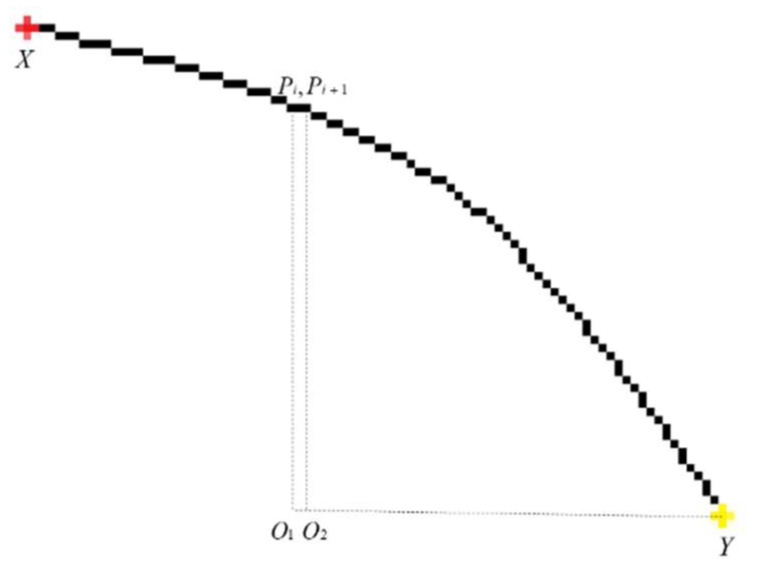
Manhattan distance position relationship of points on arcs.

The interference curve is relatively random, and the distance from the point on the arc to the end point is decreasing. As shown in Figure 4, $P_{i},P_{i+1}$, respectively, represent two consecutive points on the arc, X,Y are the two endpoints of the arc point set, respectively, and the Manhattan distance from the two points to the endpoints can be represented by $\left|\left|P_{i}O_{1}\right|+\left|O_{1}Y\right|\right|$ and $\left|\left|P_{i+1}O_{2}\right|+\left|O_{2}Y\right|\right|$, respectively. In this regard, we perform direction screening on the result of sharpness estimation to select information points. The specific direction screening is as follows:

(6) $$DirX=\left(PX_{n-2}+PX_{n-1}+PX_{n}\right)-\left(PX_{1}+PX_{2}+PX_{3}\right)$$

(7) $$DirY=\left(PY_{n-2}+PY_{n-1}+PY_{n}\right)-\left(PY_{1}+PY_{2}+PY_{3}\right)$$

(8) $$Ft\left(i\right)=\{\begin{array}{l} 1,\left(PX_{i}-PX_{i-1}\right)\times DirX<0\;or\;\left(PY_{i}-PY_{i-1}\right)\times DirY<0\\ 0,other \end{array}$$

(9) $$FalseNum=\overset{n}{\underset{i=2}{\sum }}Ft\left(i\right)$$

**Figure 5:**
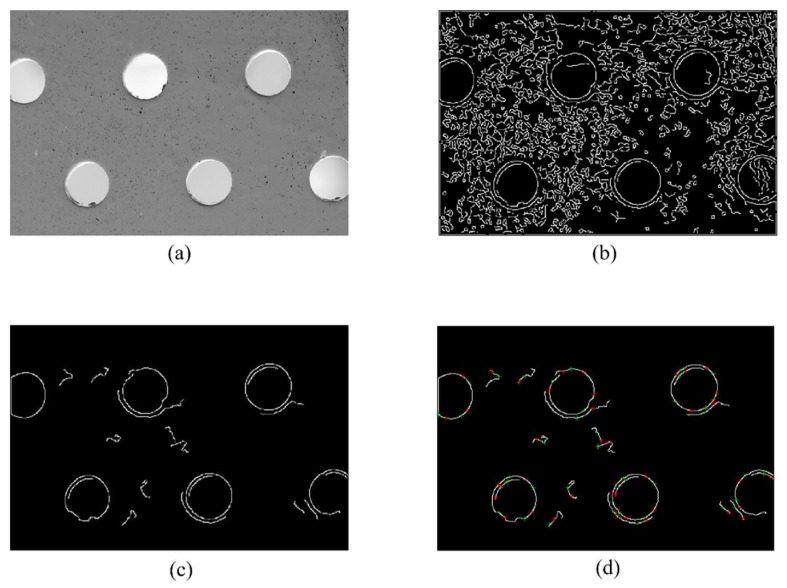
The intermediate results of our procedure are shown: (**a**) the original image; (**b**) the result of adaptive canny edge extraction; (**c**) the image after we have performed useless arc removal; (**d**) the information points we selected and used. ‘*’ and ‘+’ mark the information points.

$P_{1},P_{2},\cdots ,P_{N-1},P_{N}$ is the point on the curve. $PX_{i}\;\mathrm{and}\;PY_{i}$ represent the horizontal and vertical coordinates of the i point, respectively. DirX and DirY record the direction of the end point relative to the start point. FalseNum indicates the number of times the curve does not follow the trend. When FalseNum≥n×η (the value of η; see 4.1), these arcs are considered to be noise or unimportant information, and we remove them from the image. The results obtained by our algorithm are shown in Figure 5.

As can be seen from Figure 5, although the canny edge extraction algorithm can extract the edge of the circle very well, it is also accompanied by a large number of unimportant details and interference curves. In Figure 5c, we can see that our algorithm removes a large number of unimportant details and interfering curves, and, as shown in Figure 5d, the circle information on the picture is well preserved in information points. We validated the effect of this method on three datasets and recorded the results in Table 2.

The number of edge points in Table 2 refers to the average number of edge points obtained by the adaptive canny edge extraction algorithm, the number of information points refers to the points used to store the circle information on the image, the information compression ratio refers to the compression effect of the edge point information, the retention rate of the circle refers to the degree of the algorithm’s retention of the circle and 100% means that no circle information is lost.

It can be seen from Table 2 that our algorithm can effectively store the circle information on the image in a minimum of 0.63% of the information points, and the retention rate of the circle information can reach 100% in the process. Even on the dataset GH, which contains a lot of ambiguity, it still has 98.94% retention. This method of storing information on the image with a small number of points can not only reduce iterations but also increase the robustness of the algorithm.

It can be seen from Figure 5b that the adaptive canny edge extraction results are often accompanied by a large amount of interference. The proportion of circles in the edge points is only 11.89%. In the canny edge extraction results, only the curves where the information points are located are retained. Curves without information points will be treated as useless arcs. The performance of our algorithm on three datasets is shown in Table 3. Our method can remove, at most, 71.16% of the points, with the lowest error rate being only 0.16%. After filtering, the proportion of points on the circle on the image has increased by up to 236.20%. Our algorithm does not perform as well on the dataset Geometry as the other two datasets, mainly because the background in the dataset Geometry is relatively clean and free of ambient noise.

### 2.3. Average Sampling to Verify Candidate Circles

Traverse all the information points and select the calculation parameters ($P_{1}\left(X_{1},Y_{1}\right),\;P_{2}\left(X_{2},Y_{2}\right),\;P_{3}\left(X_{3},Y_{3}\right)$) of the three information points each time to calculate the circle parameters O(x,y,r). Then, substitute the verification parameters of the three information points for verification. If the error of the verification result is greater than max (0.5 , min(5,r/30)), the circle is considered to be a false circle. After the verification is successful, start from just above the center of the circle, and perform sampling point verification for every radian. The sampling point coordinate formula is:

(10) $$Theta=2\times P_{i}\times i\;\left(i\in 0,1,\ldots ,36\right)$$

(11) $$\{\begin{array}{c} cx=round\;\left(x+r\;\times cos\;\left(Theta\right)\right)\\ cy=round\;\left(y+r\times sin\;\left(Theta\right)\right) \end{array}$$

where cx and cy represent the horizontal and vertical coordinates of the sampling point, respectively, i represents the number of i sampling times and x,y,r refers to the circle parameter.

During the sampling process, we recorded the following parameters:

samples: Refers to the number of successful sampling verifications. If there are pixels in the 9 neighborhoods of the sampling point (when r>100, take 16 neighborhoods), we consider the sampling verification result to be true;

MaximumArc: We connect the adjacent successfully sampled points into an arc. If the interval is less than 1 sampling point, merge the two arcs, and record the longest arc;

*Left*: The radian corresponding to the left endpoint of the longest continuous arc;

*Right*: The radian corresponding to the right endpoint of the longest continuous arc;

DiscreteArc: The number of successful samplings not on the longest continuous arc.

Here, the circles are divided into two categories according to the sampling verification results: complete circles and defect circles. The following two cases are discussed to determine whether the circle parameters are candidate circles:

Complete circle judgment

The circle whose number of successful samplings and verifications is greater than $\varphi_{1}$ is considered as a candidate circle. At the same time, if the number of successful samplings and verifications exceeds $\varphi_{2}$, it is considered that the circle has reached the optimal parameters, and the information points whose Euclidean distance is less than max (0.5 , min(5,r/30)) will be deleted. According to our experimental parameters $\varphi_{1}=28$, $\varphi_{2}=33$, the running result is the best. Parameters $\varphi_{1}$ and $\varphi_{2}$ did not need to be changed in all datasets run in this paper.

Defect circle judgment

A circle with a number of successful samplings between $0.75\times \varphi_{1}$ and $\varphi_{1}$ is considered a defect circle, and we will resample the defect circle and rotate all sampling points clockwise by 5°. At the same time, compare the samples and MaximumArc of the two samples. If the difference is less than 2, it will be added to the candidate circle.

### 2.4. Find True Circles

According to [22], the following formula is used to express the overlap ratio of circles. When the overlap ratio is greater than 0.8, we consider them to be the same circle and retain the circle with better sampling results.

(12) $$Ratio\left(Cd,Ct\right)=\frac{area\left(Cd\right)\cap area\left(Ct\right)}{area\left(Cd\right)\cup area\left(Ct\right)}$$

area(Cd) and area(Ct) refer to the areas of Cd,Ct, respectively, and Ratio(Cd,Ct) refers to the overlap rate of the two circles. For the candidate circle, use the following formula to verify the true circle:

(13) $$\{\begin{array}{l} F\left(i\right)=\{\begin{array}{l} 1,\left|\sqrt{{\left(PX_{i}-cx\right)}^{2}+{\left(PY_{i}-cy\right)}^{2}}-cr)\right|<diff\\ 0,other \end{array}\\ diff=max\;\left(0.5\;,\;min(5,r/30\right)) \end{array}$$

(14) PointNum=∑F(i)

Depending on the type of the circle, there are two situations that can be discussed to determine whether a candidate circle is a true circle.

1. Complete circle judgment

If the candidate circle is marked as a complete circle and satisfies Point>2×π×r×0.8, we think it is a true circle.

2. Defect circle judgment

If the candidate circle is marked as a defective circle, we perform defect circle verification. First, Circles that do not satisfy Point>2×π×r×0.6 will be excluded. Then, to prevent random noise from interfering with the average sampling verification, we use ArmSum and DisSum to record and verify the longest arc and discrete arc of the defect circle, respectively. When the number of points of the longest arc and the number of points of the non-longest arc satisfy formula (19), we consider the defective circle to be a true circle.

(15) $$angle\left(i\right)=\arctan(\frac{PY_{i}-cy}{PX_{i}-cx}\times \frac{180}{\pi })$$

(16) $$mark\left(i\right)=\{\begin{array}{l} 1,left\leq angle\left(i\right)\leq right\\ 0,other \end{array}$$

(17) $$\{\begin{array}{l} \mathrm{ArcDet}\left(i\right)=\{\begin{array}{l} 1,mark\left(i\right)=1\;and\left|\sqrt{{\left(PX_{i}-cx\right)}^{2}+{\left(PY_{i}-cy\right)}^{2}}-cr)\right|<diff\\ 0,other \end{array}\\ ArmSum=\sum \mathrm{ArcDet}\left(i\right) \end{array}$$

(18) $$\{\begin{array}{l} \mathrm{DisDet}\left(i\right)=\{\begin{array}{l} 1,mark\left(i\right)=0and\left|\sqrt{{\left(PX_{i}-cx\right)}^{2}+{\left(PY_{i}-cy\right)}^{2}}-cr)\right|<diff\\ 0,other \end{array}\\ DisSum=\sum \mathrm{DisDet}\left(i\right) \end{array}$$

(19) $$\{\begin{array}{l} ArmSum>2\times \pi \times r\times \frac{\left(right-left\right)}{36}\\ DisSum>2\times \pi \times r\times DiscreteArc \end{array}$$

where $PX_{i}\;\mathrm{and}\;PY_{i}$ are the abscissa and ordinate of the i point in the edge image, respectively, and cx,cy,r are the abscissa and radius of the center of the candidate circle.

## 3. Proposed Circle Detection Algorithm

This section shows the flow and pseudocode of our proposed circle detection algorithm. Our proposed circle detection algorithm can be described as follows:

Step 1. Input a picture and perform Gaussian filtering on it, along with adaptive canny edge extraction;

Step 2. Perform arc extraction on the result of canny edge detection, connect adjacent points to an arc and merge the arc point sets whose endpoints are not more than one pixel apart;

Step 3. Click on the arc to estimate the sharpness, and save the arc segment whose length is greater than L;

Step 4. According to the direction screening, select the information points in the arc segments selected by the sharpness estimation; then, filter out the information points and remove the useless arcs on the picture;

Step 5. If all the points of the information point are judged, or the number of information points is less than 3, we jump to Step 6. Otherwise, the circle parameters are calculated for the points in the candidate field in turn, and the candidate circle is determined; then, jump to Step 5;

Step 6. Delete the duplicate circles in the candidate circles;

Step 7. The circle detection algorithm ends, and, finally, the detection results are verified.

The proposed algorithm can also be expressed in pseudocode, as follows Algorithm 1:
**Algorithm 1**: Proposed Circle Detection AlgorithmInput: Grayscale imageOutput: Detected circles 1: Initialization parameters 2: Gaussian filter for image 3: Adaptive canny edge extraction for images 4: Sharpness extraction from images 5:   **if** Direction filter passed **then** 6:       add to information point collection Ω 7:   **end if** 8: Image cleanup 9: **for** ai∈Ω**do** 10:     **for** aj∈Ω**do** 11:         **for** ak∈Ω**do** 12:             **if** i==j or i==j or j==k **then** 13:                 continue; 14:           **end if** 15:           Select three information points to calculate circle parameters 16:           **if** Information point verification parameter verification failed **then** 17:               continue; 18:           **end if** 19:           Perform average sampling verification on the circle parameters 20:           **if** not **then** 21:               continue; 22:           **end if** 23:           **if** The number of successful sampling is greater than 33 times **then** 24:               delete information points on the circle 25:           **end if** 26:       **end for** 27:   **end for** 28: **end for** 29: Remove duplicate circles in candidate circles 30: Verification of candidate circles (Section 2.4) to find true circles 31: **if** not **then** 32:       continue; 33: **end if**

## 4. Experiments and Results Analysis

In this chapter, we compare the proposed algorithm with five other algorithms. The first is the voting-based RHT [8] algorithm, the second is the sampling-based detection RCD [9] algorithm, the third is the Jiang [11] proposed optimization algorithm, which we refer to as Jiang for short, the fourth is the curvature-based CACD [17], the fifth is the middle-time Wang’s algorithm and the last is our algorithm. In order to unify the standard, it is stipulated here that the proportion of the occluded part of the circle cannot exceed 0.4 times the circumference of the circle. All the above algorithms were executed in MATLAB R2019b in order to exclude language interference on the running time, and they were all run on the same computer using an Intel Corei5 CPU 2.90 GHz and 8 GB RAM. For the objectivity and accuracy of the experiment, the following four indicators will be used to measure: Precision, Recall, F-measure and Time. Time refers to the time from inputting the picture to outputting all of the found circle information.

(20) $$Precision=\frac{TP}{TP+FP}$$

(21) $$Recall=\frac{TP}{TP+FN}$$

(22) $$F-measure=2\times \frac{Precision\times Recall}{Precision+Recall}$$

The experiments refer to the verification indicators used in the literature [16,19,23,24,25,26], and when the coincidence rate of the circles is not lower than 0.8, they are considered to be the same circle. Treat it as a true positive (TP); otherwise, it is a false positive (FP), and the ground truth that is not correctly identified is treated as a false negative (FN). Formula (12) is used to define the overlap ratio between circles Cd and Ct: 

The test images in this paper are mainly from our dataset and two public datasets available on the internet: 

Dataset Geometry. It is a dataset containing complex curves and consists of 13 images. Large-size pictures, complex curve interaction and large radius changes bring difficulties to the measurement of circles.

Dataset GH. It is a complex dataset from [21] consisting of 257 real-world gray images. Blurred edges, large changes in radius and occlusions make measurements inconvenient.

Dataset PCB. It is an industrial dataset from [20] which contains 100 printed circuit board images. A large amount of noise and a large number of concentric circles with blurred edges make the measurement difficult.

### 4.1. Threshold Analysis

**Figure 6:**
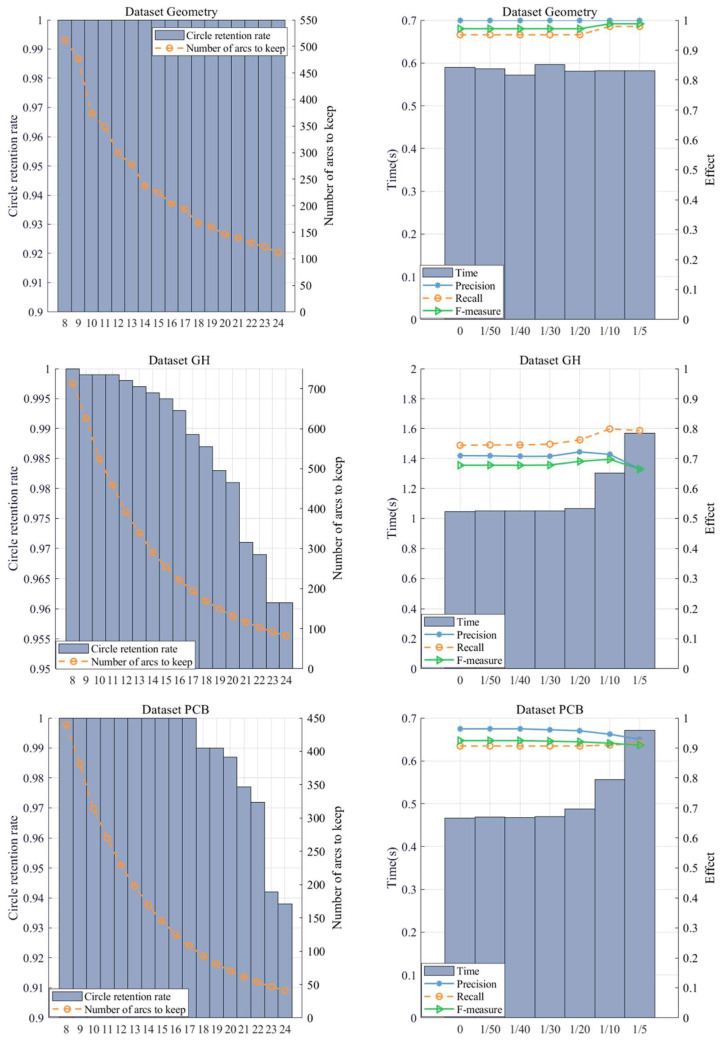
On the left is the threshold and intermediate result for parameter L, and on the right is the threshold and final result for parameter η.

Our algorithm mainly involves two parameters: L and η. Due to the complexity of the images, it is impossible to fix all parameters for optimal performance. Furthermore, the relationship between the parameters L and η is “ $L\times X=Z_{1},\;\eta \times Z_{1}=Z$”. We can obtain the optimal intermediate result $Z_{1}$ by adjusting the parameter L. On this basis, we adjust η to obtain the final result. The process of adjusting the parameter L to obtain the optimal intermediate result $Z_{1}$ and adjusting the parameter η to obtain the final result is shown in Figure 6.

In the sharpness estimation stage, the parameter L is used to filter the arc, which is very important for the selection of subsequent information points. If L is too small, the number of arcs will increase, and the algorithm efficiency will decrease. If L is too large, part of the circle information will be lost. We suggest that the value should be appropriately increased in images with sharp edges and should be appropriately decreased in real images.

In the screening stage of information points, parameter η represents the allowable error rate. With the increase in η, the number of information points increases. The Recall rate will increase relatively, while the Precision will decrease accordingly, and the Time will also increase. In a real image, due to the interference of noise, the edge lines of the circle will be disturbed, so this parameter needs to be appropriately increased, and on an ideal picture with a clear background, this parameter can be appropriately decreased.

### 4.2. Performance Comparison

#### 4.2.1. Dataset Geometry

**Figure 7:**
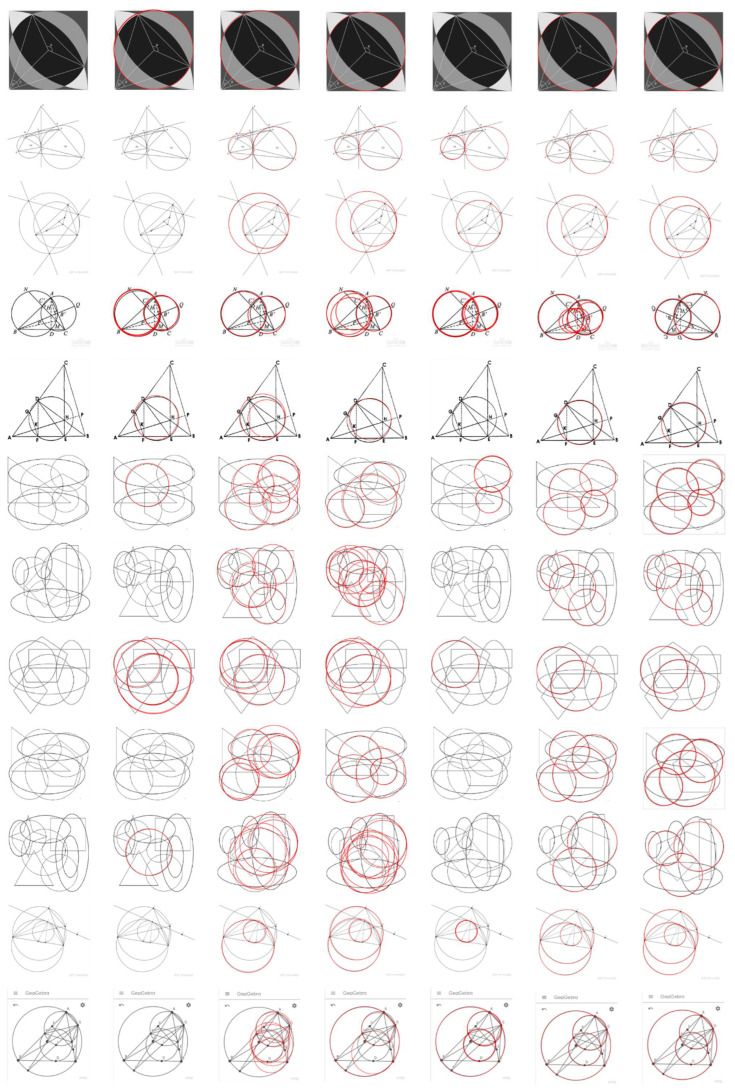
Circle detection results on the dataset Geometry. From the left to right columns: input image, RHT, RCD, CACD, Jiang, Wang and ours. As can be seen, the proposed method obtains better performance than others.

We first report the detection results for the dataset Geometry in Figure 7. The F-measure and Time of the six algorithms on each image are shown in detail in Table 4 and Table 5. Finally, the run results for the entire dataset are summarized in Table 6.

As can be seen from the chart, the RHT algorithm has many missed detections. In contrast, RCD has better performance than RHT. Jiang’s method has a better performance in terms of Recall and F-measure, but the running time has increased, mainly because the number of interference points in the area of differential evidence collection is large. The CACD algorithm does not perform well in the dataset Geometry. The complex curve interleaving makes circle fitting difficult, and with the change in image size (such as the 3rd and 11th pictures in Figure 7; the size is 1326 × 1536), the running time of the program is greatly increased. Wang’s algorithm also performed very well on this dataset, with only three images not correctly detected. Our algorithm has achieved the best results on this dataset, and there is a missed detection in the seventh picture because the information points in this part are relatively dense and our algorithm mistakenly deletes some information points on the candidate circle during verification.

#### 4.2.2. Dataset GH

**Figure 8:**
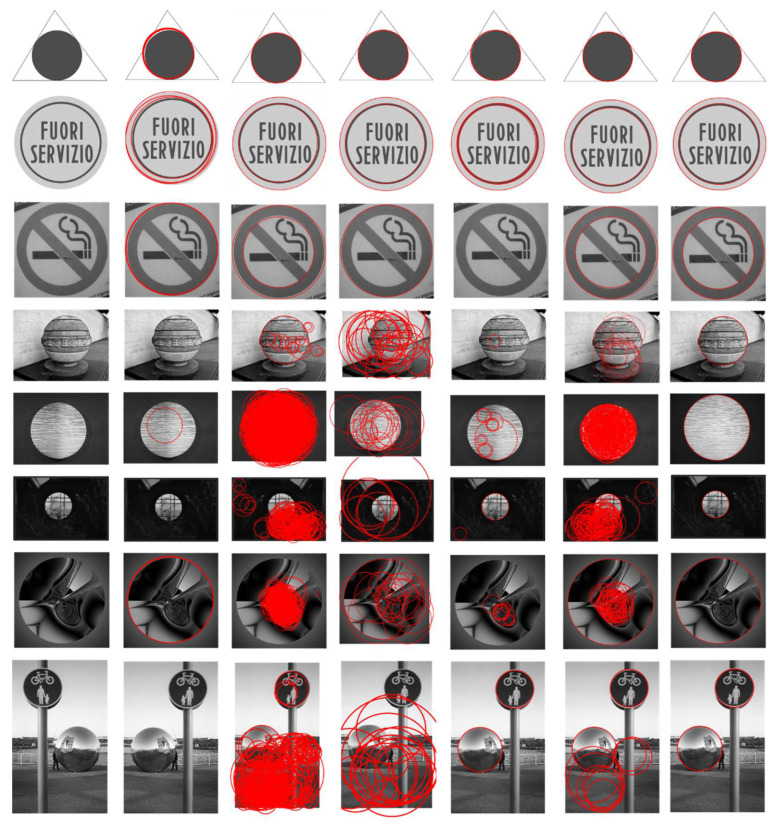
Circle detection results on the dataset GH, which is widely used by other algorithms. From the left to right columns: input image, RHT, RCD, CACD, Jiang, Wang and ours. As can be seen, the proposed method obtains better performance than the others.

Next, we report the detection results for the dataset GH. Combining the data in Figure 8 and Table 7, it can be seen that RHT does not perform well on noisy images. RCD has a high Recall but cannot ensure a high Precision. Jiang’s algorithm has improved time and accuracy compared to RCD. The CACD algorithm performs well in most real images without complex texture interference, but when there are many textures and the image is large, the time is often very slow, and there will be missed detections and false detections. Wang’s algorithm performed poorly on this dataset. This is because, in real images, a large number of dense interference points make the algorithm detect a large number of false circles that cannot be eliminated. Our algorithm removes many interfering edges before detection, which not only speeds up the running time but also reduces their interference to the circle validation stage, improves precision and recall and maintains good performance for most images in the dataset GH.

#### 4.2.3. Dataset PCB

**Figure 9:**
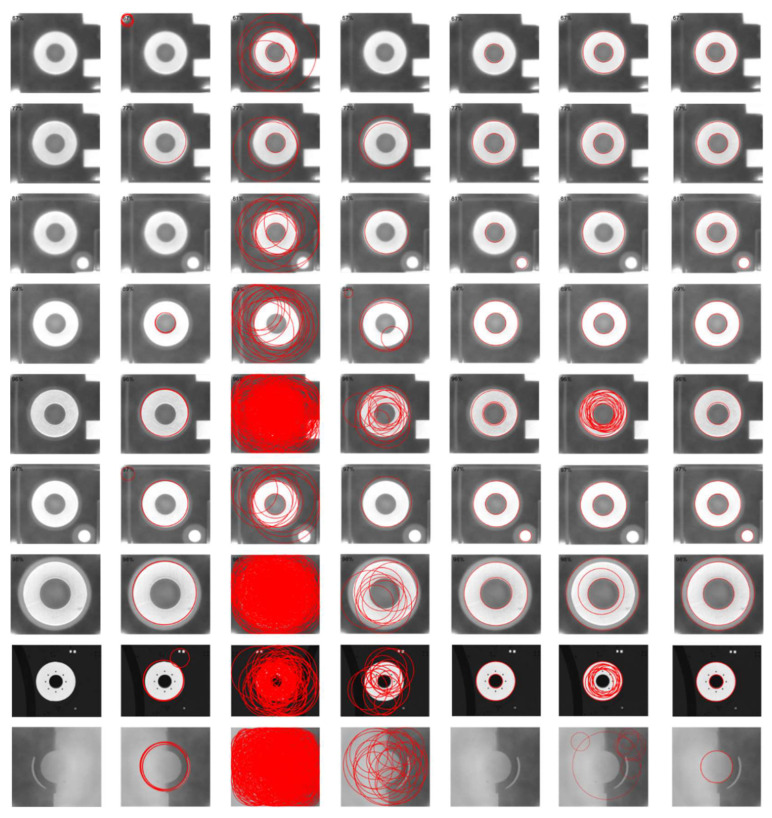
Circle detection results on the dataset PCB, which is widely used by other algorithms. From the left to right columns: input image, RHT, RCD, CACD, Jiang, Wang and ours. As can be seen, the proposed method obtains better performance than the others.

Finally, we report the detection results for the dataset PCB. Combining the data in Figure 9 and Table 8, it can be seen that, under the noise interference, the RHT algorithm has some defects, including false detection, a low recall and a slower running speed. The RCD algorithm still shows a low precision and a high recall. A large amount of noise interference reduces the probability that the sampling points are on the same circle and also increases the possibility of false detection. Jiang’s algorithm maintains good performance when the edge is clear and improves the accuracy, but, as the blurring of the image increases, the number of points in the difference evidence collection area increases sharply, which will slow down the running speed and reduce the accuracy. CACD works well on this dataset, but when the image is too blurry and the edge extraction algorithm cannot extract continuous edges, CACD will not be able to detect the corresponding circle, such as the seventh image in the figure below. Wang’s algorithm outperformed Jiang’s algorithm on this dataset and performed similarly to CACD, mainly because, on this dataset, there are relatively few interference points. When the picture is blurred, the algorithm also cannot find the circle correctly. Our algorithm does not rely on continuous edge extraction and eliminates useless arcs. It not only runs faster but also prevents subtle errors and noise from interfering with the results.

### 4.3. Discussion

As can be seen from Table 6, Table 7 and Table 8, our proposed method has some advantages over other methods. Compared with the above methods, our method can more effectively streamline the circle information on the image to improve the detection speed. Compared with Wang’s method, our algorithm is more practical. In complex graphs with clean backgrounds, Wang’s algorithm is able to maintain good performance. However, in images with more noise, the performance of Wang’s algorithm degrades rapidly. Compared to CACD, our method does not need to iterate over a large number of radius layers and remains stable when the image size is large. On the dataset GH and the dataset PCB, our algorithm exhibits different characteristics. The Recall on the dataset GH is higher than the Precision. This is due to the large amount of interference in the dataset GH, which brings difficulties for circle validation. The Precision on the dataset PCB is higher than the Recall; this is because a large amount of blur makes information point compression troublesome, and our algorithm inevitably loses some circle information. F-measure is a combination of precision and recall, and we show, in Section 4.1, our process of adjusting the image to achieve optimal parameters during the threshold analysis stage of the parameters.

## 5. Conclusions

This paper proposes a fast circle detection algorithm based on information compression and analyzes its performance. The algorithm achieves good performance through four stages of image preprocessing, information compression and screening, average sampling to verify candidate circles and finding true circles. (1) In terms of detection speed, we introduce an idea of information compression: compressing the circle information on the image into a few information points and using an average sampling algorithm with a time complexity of O(1) to verify the candidate circle, which effectively speeds up the speed of the algorithm. (2) In terms of detection accuracy, our algorithm removes interference information and effectively eliminates the false detections caused by small edges on the image.

We tested three datasets. The results show that our method can compress the circle information on the image to the lowest 0.63% points and remove the highest 71.16% of the interference points in the image, with the lowest false deletion ratio being only 0.16%. Our algorithm outperforms RHT, RCD, Jiang and CACD in terms of Precision, Recall, F-measure and Time.

## Figures and Tables

**Table 1:** Summary of previous work.

Circle Detection Algorithm	—Previous Work
HT	HT transforms the original image into the parameter space and finds the true circle by voting [7].
RHT	RHT finds the true circle by randomly picking three points to vote [8].
RCD	The RCD randomly samples four points; three points are used to determine the circle parameters, and the fourth point is used for verification [9].
Jiang’s algorithm	Jiang uses difference region sampling to improve sampling accuracy and find true circles [11].
Wang’s algorithm	Wang uses the sub-pixel algorithm to regularly sample the gradient of the pixel points in order to find the true circle [12].
Le’s algorithm	Le obtains the circle parameters by the least squares method [14].
Zhen’s algorithm	Zhen finds the true circle by calculating the radius of the curvature of the arc [17].

**Table 2:** The performance of our algorithm on the dataset.

	Number of Edge Points	Number of Information Points	Information Compression Ratio	Retention Rate of the Circle
Geometry	21,969	238.25	1.12%	100.00%
GH [21]	21,339.27	166.73	1.00%	98.94%
PCB [21]	13,911.45	86.09	0.63%	100.00%

**Table 3:** The result of useless arc removal.

	Geometry	GH [21]	PCB [21]
Number of edge points	21,969	21,339.27	13,911.45
Number of points after filtering	16,487.50	9895.81	3966.84
Clear rate	24.36%	51.38%	71.16%
Percentage of points on the circle among edge points	24.66%	11.89%	11.97%
The percentage of points on the circle after clearing	30.35%	23.44%	38.42%
Probability boost	26.95%	135.47%	236.20%
Mistaken deletion ratio	0.93%	0.35%	0.16%

**Table 4:** F-measure of RHT, RCD, CACD, Jiang, Wang and our method in the dataset Geometry.

	RHT	RCD	Jiang	CACD	Wang	Our
1	1	1	1	0	1	1
2	0	1	1	0.8	1	1
3	0	0.667	1	0.667	1	1
4	0.8	0.667	0.667	0.571	0.5	1
5	1	0	1	0	1	1
6	0.4	0.571	0.5	0.571	1	1
7	0	0.8	0.222	0	0.857	0.857
8	0	1	0.667	0.667	1	1
9	0	0.4	0.571	0	1	1
10	0	0.4	0.095	0	0.8	1
11	0	0.5	1	0.4	1	1
12	0	0.444	0.667	0.75	1	1

**Table 5:** Time of RHT, RCD, CACD, Jiang, Wang and our method in the dataset Geometry (unit: s).

	RHT	RCD	Jiang	CACD	Wang	Our
1	22.25	6.50	4.06	2.14	1.82	0.13
2	37.46	6.50	8.07	16.90	1.53	0.36
3	39.64	6.71	23.14	494.15	1.63	2.06
4	16.90	6.49	2.33	0.69	1.58	0.16
5	35.72	6.45	7.66	2.65	1.74	0.28
6	32.53	6.30	8.42	1.39	1.56	0.20
7	35.73	6.30	15.84	1.42	1.53	0.25
8	32.64	6.28	9.36	2.10	1.53	0.19
9	33.77	6.35	3.86	1.72	1.56	0.23
10	33.99	6.26	3.46	1.67	1.70	0.25
11	36.23	6.51	16.55	202.71	1.64	2.40
12	32.17	6.31	11.48	9.01	1.80	0.58

**Table 6:** Result of RHT, RCD, CACD, Jiang, Wang and our method in the dataset Geometry.

	Precision	Recall	F-Measure	Time (s)
RHT	0.31	0.27	0.27	32.42
RCD	0.69	0.66	0.62	6.41
CACD	0.40	0.40	0.37	61.38
Jiang	0.68	0.79	0.70	9.52
Wang	0.94	0.95	0.93	1.64
Our	1.00	0.98	0.99	0.59

**Table 7:** Results on the dataset GH.

	Precision	Recall	F-Measure	Time (s)
RHT	0.14	0.13	0.13	11.07
RCD	0.03	0.69	0.04	6.74
CACD	0.5	0.73	0.54	4.33
Jiang	0.18	0.32	0.20	2.15
Wang	0.32	0.51	0.29	1.65
Our	0.71	0.80	0.70	1.30

**Table 8:** Result on the dataset PCB.

	Precision	Recall	F-Measure	Time (s)
RHT	0.35	0.40	0.30	15.11
RCD	0.19	0.65	0.25	6.35
CACD	0.65	0.80	0.69	2.09
Jiang	0.31	0.43	0.33	2.85
Wang	0.69	0.77	0.66	1.59
Our	0.97	0.91	0.93	0.46

## Data Availability

Not applicable.
